# Supplementary material for: Potentiating effect of reovirus on immune checkpoint inhibition in microsatellite stable colorectal cancer
Source: Front Oncol. 2022 Oct 25;12:1018767. doi: 10.3389/fonc.2022.1018767 (PMC9642964; doi:10.3389/fonc.2022.1018767)
Supplement: Supplementary Figure 1 — Individual growth arrest pattern of the 13 cell lines upon reovirus treatment. MTT assay revealed that reovirus treatment of 5 MOI for 24 hours induced significant growth arrest in all 13 CRC cell lines studied. [file Presentation_1.pdf]

Potentiating effect of reovirus on immune checkpoint inhibition in microsatellite stable colorectal cancer

Supplementary Materials

Supplementary Table 1.

	Cell Line	MMR status	*KRAS/Kras* status
Human	HCT116	MSI	MUT
	Hke3	MSI	WT
	LIM2405	MSI	WT
	HT29	MSS	WT
	SW837	MSS	MUT
	SW620	MSS	MUT
	Caco2	MSS	WT
Mouse	MC38	MSI	WT
	CT26	MSS	MUT

*MSI – microsatellite unstable, MSS – microsatellite stable, MUT – mutant, WT – wild type

Supplementary Table 2.

Application	Target	Clone	Species	Dilution	Source Company, Cat#
Flow cytometry	CD274 (PD-L1) - PE	29E.2A3	Mouse	1:20	BioLegend, 329706
	CD273 (PD-L2) - APC	24F.10C12	Mouse	1:20	BioLegend, 329608
	CD133 - PE	TMP4	Mouse	1:20	Invitrogen, 12-1338-42
	CD44 - APC	IM7	Rat	1:20	Invitrogen, 17-0441-82
	CD24 – PE-Cy7	SN3 A5-2H10	Mouse	1:20	Invitrogen, 25-0247-42
	CD326 (EpCAM) – AF700	AUA1	Mouse	1:40	Novus Biologicals, NB600-1182
	CD45 – BV605	HI30	Mouse	1:50	BD Biosciences, 564047
	CD45 – BV421	HI30	Mouse	1:70	BD Biosciences, 563879
	ROR2 – AF488	231509	Mouse	1:40	Novus Biologicals, FAB20641G
	CD8a - FITC	53-6.7	Rat	1:50	BD Biosciences, 553031
	CD8a – BUV395	53-6.7	Rat	1:50	BD Biosciences, 563786
	CD11b – AF700	M1/70	Rat	1:100	Invitrogen, 56-0112-82
	CD11b – BV605	M1/70	Rat	1:70	BioLegend, 101257
	CD11c- FITC	HL3	Hamster	1:50	BD Biosciences, 557400
	CD4 - APC	RM4.5	Rat	1:70	BD Biosciences, 553051
	CD4 – PE-Cy5	RM4.5	Rat	1:50	BD Biosciences, 553050
	CD3 – PE-Cy7	17A2	Rat	1:50	BD Biosciences, 560591
	FoxP3 – PE-Cy5	150D	Mouse	1:50	BioLegend, 320008
	NK-1.1 – PE-Dazzle 594	PK136	Mouse	1:50	BioLegend, 108748
	NK-1.1 – BV605	PK136	Mouse	1:50	BioLegend, 108753
	T-Bet - PE	4B10	Mouse	1:50	BD Biosciences, 561265
	GATA3 – BV421	L50-823	Mouse	1:50	BD Biosciences, 563349
	TNF-α – PerCP-Cy5.5	MP6-XT22	Rat	1:50	BioLegend, 506322
	CD107a - FITC	1D4B	Rat	1:70	BioLegend, 121606
	Tim-3 - APC	5D12	Mouse	1:100	BD Biosciences, 567164
	IFN-γ – PE-Dazzle 594	XMG1.2	Rat	1:50	BioLegend, 505846
	PD-1 - PE	29F.1A12	Rat	1:70	BioLegend, 135206
	MHC-I – PerCP-Cy5.5	M5/114.15.2	Rat	1:50	BD Biosciences, 562363
	MHC-II – AF700	M5/114.15.2	Rat	1:70	Invitrogen, 56-5321-82
	Ly-6G - APC	RB6-8C5	Rat	1:50	BD Biosciences, 553129
	Ly-6C – PE-Cy7	RB6-8C5	Rat	1:50	BD Biosciences, 565033
	F4/80 – PE-Cy5	BM8	Rat	1:100	BioLegend, 123112
	CD206 (MMR) - PE	C068C2	Rat	1:50	BioLegend, 141706
	NLRP3 – AF350	768319	Mouse	1:50	R&D Systems, IC7578U
	CD283 (TLR3) - PE	TLR3.7	Mouse	1:50	Invitrogen, 12-9039-82

Application	Target	Clone	Species	Dilution	Source Company, Cat#
Immunohistochemistry	**Ki67**	**Polyclonal**	**Rabbit**	**1:400**	**Bethyl Labs, IHC-00375**
	Cleaved Caspase 3	Polyclonal	Rabbit	1:100	Invitrogen, PA5-114687
	TUNEL assay kit	ApopTag® Plus Peroxidase In Situ Apoptosis Kit, Millipore Sigma S7101			
	CD3	CD3-12	Rat	1:200	Abcam, ab11089
	CD8a	4SM16	Rat	1:100	Invitrogen, 14-0195-82
	Granzyme B	Polyclonal	Rabbit	1:100	Invitrogen, PA5-13518
Immunofluorescence	PD-L1 – DyLight 488	MIH5	Rat	1:1000	Novus Biologicals, NBP1-43262G
Western blotting	PD-L1	E1L3N	Rabbit	1:1000	Cell Signaling, 13684
	PD-1	D7D5W	Rabbit	1:1000	Cell Signaling, 84651
	RIG-I	D14G6	Rabbit	1:1000	Cell Signaling, 3743
	MDA5	D74E4	Rabbit	1:1000	Cell Signaling, 5321
	TLR3	TLR3.7	Mouse	1:500	Santa Cruz, sc-32232
	NLRP3	D4D8T	Rabbit	1:1000	Cell signaling, 15101
	PKR	B-10	Mouse	1:400	Santa Cruz, sc-6282
	B-Actin	AC-74	Mouse	1:10000	Sigma-Aldrich, A2228

Target	Forward	Reverse
Human PD-L1 (CD274)	GCTATGGTGGTGCCGACTAC	CAGATGACTTCGGCCTTGGG
Human PD-L2 (PDCD1LG2)	GAACCCAGGACCCATCCAAC	AGACCACAGGTTCAGATAGCAC
Human IFN-gamma	GCATCGTTTTGGGTTCTCTTGGCTGTTACTGC	CTCCTTTTTCGCTTCCCTGTTTTAGCTGCTGG
Human IRF-1	CGAATCGCTCCTGCAGCAGA	GCCCAGCTCCGGAACAAACA
Human TNF-alpha	GCCATTGGCCAGGAGGGC	CGCCACCACGCTCTTCTG
Human IL-1 beta	AAGCTGATGGCCCTAAACAG	AGGTGCATCGTGCACATAAG
Human Beta-Actin	CATTGTGATGGACTCCGGAGACGG	CATCTCCTGCTCGAAGTCTAGAGC
Mouse PD-L1 (CD274)	CCAGCCACTTCTGAGCATGA	AAACATCATTCGCTGTGGCG
Mouse PD-L2 (PDCD1LG2)	CTGCCGATACTGAACCTGAGC	GCGGTCAAAATCGCACTCC
Mouse SHP2 (PTPN11)	AACGTCAAAGAAAGTGCCGC	CTCTCTGTGTTTCCTTGTCCGA
Mouse NFATc2	CAGATCACAGCACACGGT	GATCGGTTCTTCTCGGTCC
Mouse NLRP3	ATTACCCGCCCGAGAAAGG	TCGCAGCAAAGATCCACACAG
Mouse GAPDH	AAGAGGGATGCTGCCCTTAC	TCTACGGGACGAGGAAACAC

Supplementary Table 3.

Group	Experimental Set-up	Group
1	CRC cells + 5X PBMC + BacMam Green + Cytotox Red + PBS	Control
2	CRC cells + 5X PBMC + BacMam Green + Cytotox Red + 2 MOI Reovirus	Reovirus
3	CRC cells + 5X PBMC + BacMam Green + Cytotox Red + 2 nm Nivolumab	Anti-human-PD-1
4	CRC cells + 5X PBMC + BacMam Green + Cytotox Red + 2 MOI Reovirus + 2 nm Nivolumab	Combination

Supplementary Figure 1.

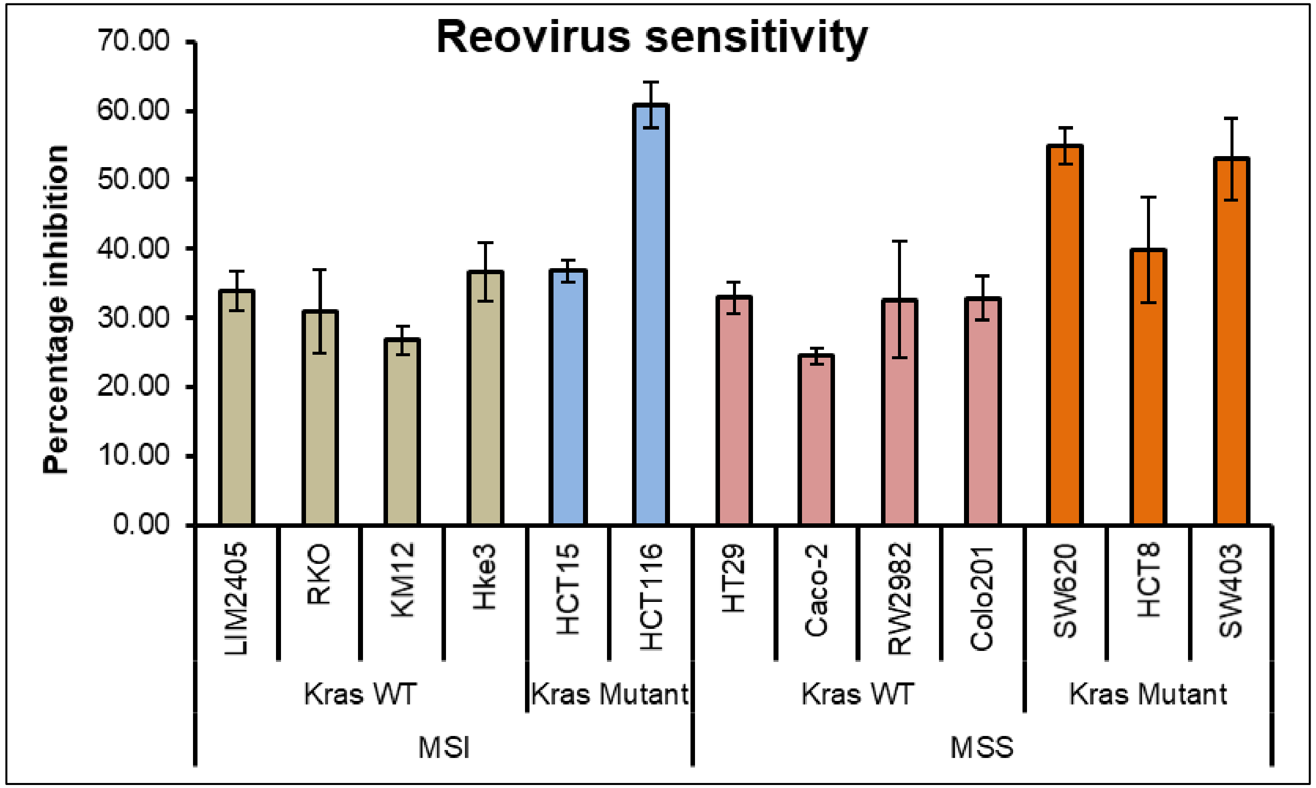

Supplementary Figure 2.

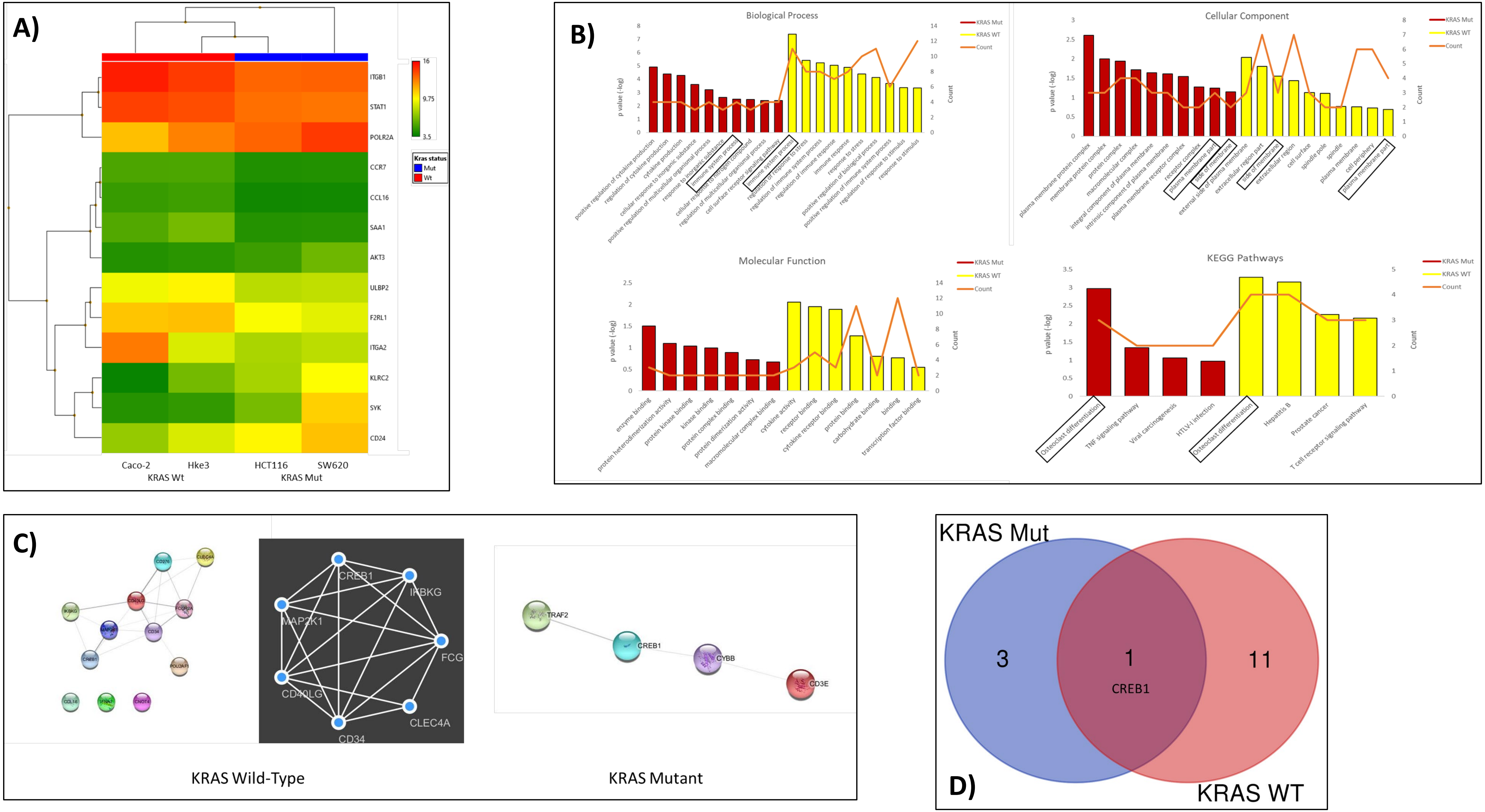

Supplementary Figure 3.

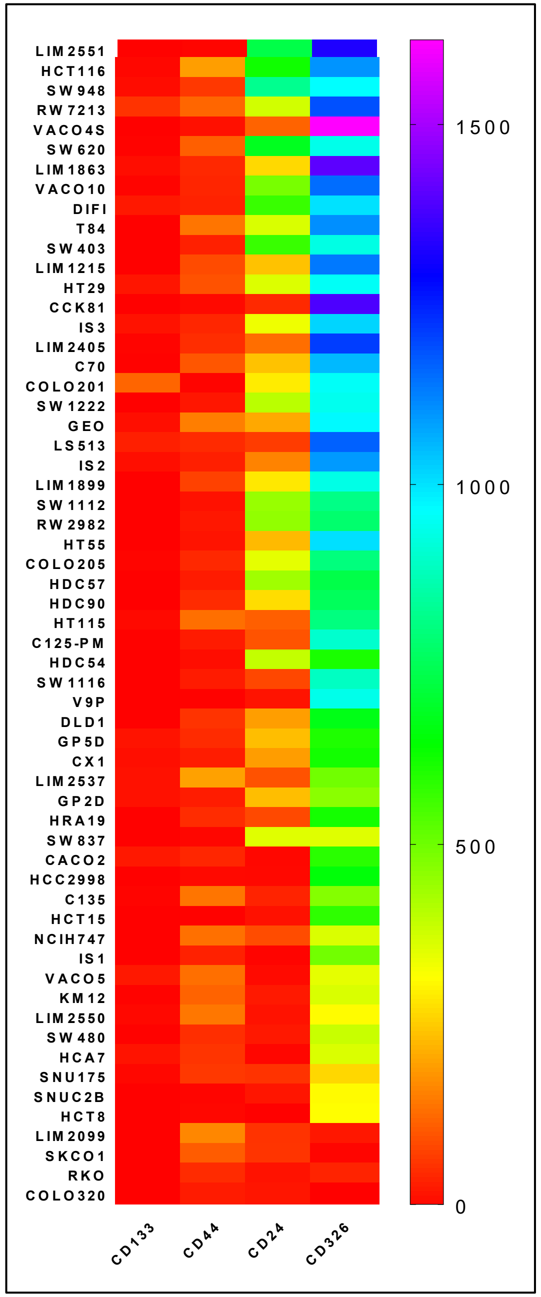

Supplementary Figure 4.

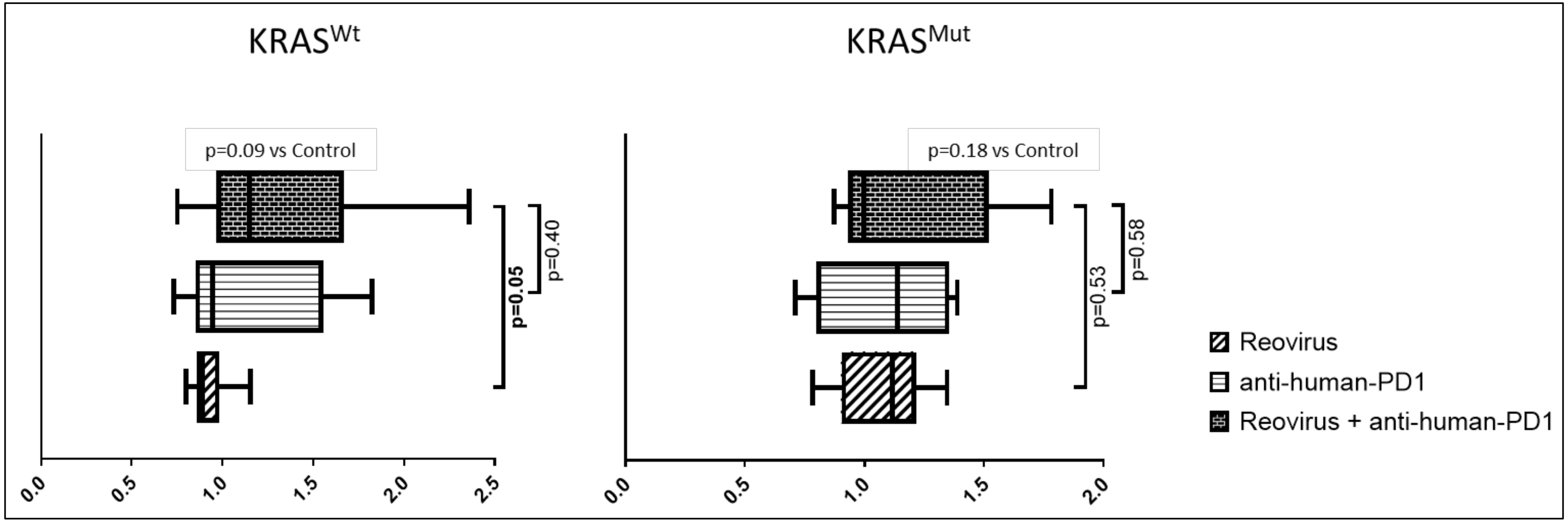

Supplementary Figure 5.

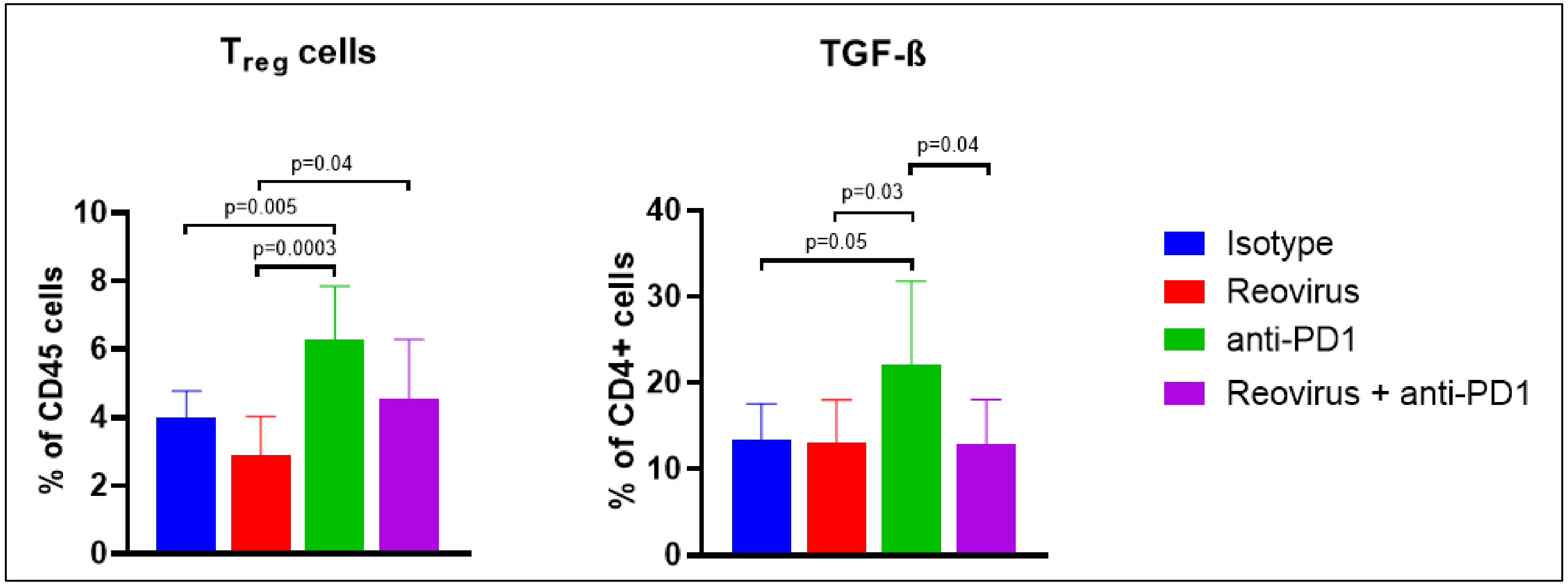

Supplementary Figure 6.

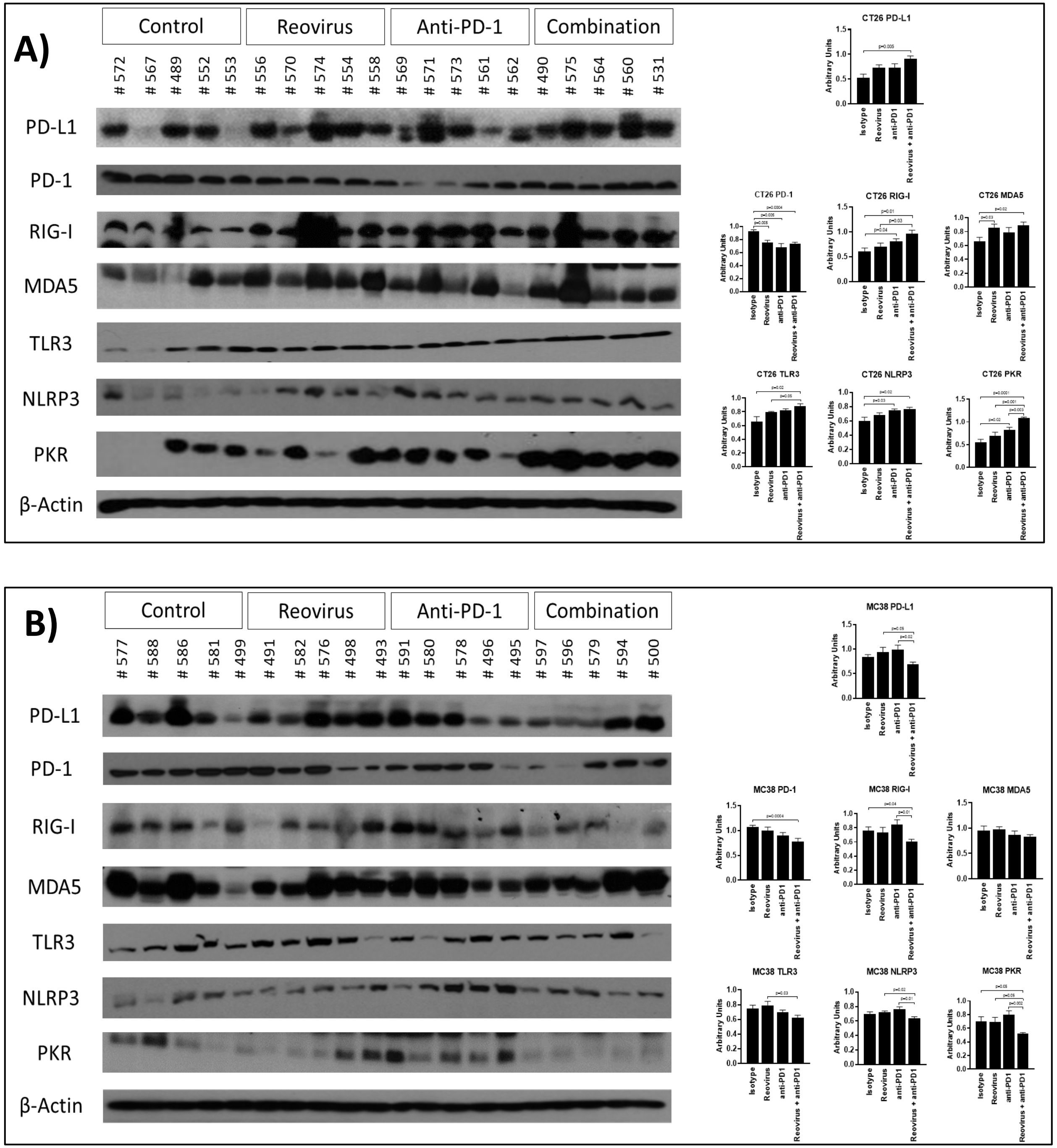

Supplementary Movies/Videos

(HT29.mp4 and LIM2405.mp4 movies are included as separate files)
